# Molecular mechanism of azithromycin resistance among typhoidal *Salmonella* strains in Bangladesh identified through passive pediatric surveillance

**DOI:** 10.1371/journal.pntd.0007868

**Published:** 2019-11-15

**Authors:** Yogesh Hooda, Mohammad S. I. Sajib, Hafizur Rahman, Stephen P. Luby, Joseph Bondy-Denomy, Mathuram Santosham, Jason R. Andrews, Samir K. Saha, Senjuti Saha

**Affiliations:** 1 Child Health Research Foundation, Department of Microbiology, Dhaka Shishu Hospital, Dhaka, Bangladesh; 2 MRC Laboratory of Molecular Biology, Cambridge, United Kingdom; 3 Division of Infectious Diseases and Geographic Medicine, Stanford University School of Medicine, Stanford, California, United States of America; 4 Department of Microbiology and Immunology, University of California, San Francisco, San Francisco, California, United States of America; 5 Quantitative Biosciences Institute, University of California, San Francisco, San Francisco, California, United States of America; 6 Department of International Health, Johns Hopkins Bloomberg School of Public Health, Baltimore, Maryland, United States of America; 7 Bangladesh Institute of Child Health, Dhaka Shishu Hospital, Dhaka, Bangladesh; Johns Hopkins Bloomberg School of Public Health, UNITED STATES

## Abstract

**Background:**

With the rise in fluoroquinolone-resistant *Salmonella* Typhi and the recent emergence of ceftriaxone resistance, azithromycin is one of the last oral drugs available against typhoid for which resistance is uncommon. Its increasing use, specifically in light of the ongoing outbreak of extensively drug-resistant (XDR) *Salmonella* Typhi (resistant to chloramphenicol, ampicillin, cotrimoxazole, streptomycin, fluoroquinolones and third-generation cephalosporins) in Pakistan, places selective pressure for the emergence and spread of azithromycin-resistant isolates. However, little is known about azithromycin resistance in *Salmonella*, and no molecular data are available on its mechanism.

**Methods and findings:**

We conducted typhoid surveillance in the two largest pediatric hospitals of Bangladesh from 2009–2016. All typhoidal *Salmonella* strains were screened for azithromycin resistance using disc diffusion and resistance was confirmed using E-tests. In total, we identified 1,082 *Salmonella* Typhi and Paratyphi A strains; among these, 13 strains (12 Typhi, 1 Paratyphi A) were azithromycin-resistant (MIC range: 32–64 μg/ml) with the first case observed in 2013. We sequenced the resistant strains, but no molecular basis of macrolide resistance was identified by the currently available antimicrobial resistance prediction tools. A whole genome SNP tree, made using RAxML, showed that the 12 Typhi resistant strains clustered together within the 4.3.1.1 sub-clade (H58 lineage 1). We found a non-synonymous single-point mutation exclusively in these 12 strains in the gene encoding AcrB, an efflux pump that removes small molecules from bacterial cells. The mutation changed the conserved amino acid arginine (R) at position 717 to a glutamine (Q). To test the role of R717Q present in azithromycin-resistant strains, we cloned *acrB* from azithromycin-resistant and sensitive strains, expressed them in *E*. *coli*, Typhi and Paratyphi A strains and tested their azithromycin susceptibility. Expression of AcrB-R717Q in *E*. *coli* and Typhi strains increased the minimum inhibitory concentration (MIC) for azithromycin by 11- and 3-fold respectively. The azithromycin-resistant Paratyphi A strain also contained a mutation at R717 (R717L), whose introduction in *E*. *coli* and Paratyphi A strains increased MIC by 7- and 3-fold respectively, confirming the role of R717 mutations in conferring azithromycin resistance.

**Conclusions:**

This report confirms 12 azithromycin-resistant *Salmonella* Typhi strains and one Paratyphi A strain. The molecular basis of this resistance is one mutation in the AcrB protein at position 717. This is the first report demonstrating the impact of this non-synonymous mutation in conferring macrolide resistance in a clinical setting. With increasing azithromycin use, strains with R717 mutations may spread and be acquired by XDR strains. An azithromycin-resistant XDR strain would shift enteric fever treatment from outpatient departments, where patients are currently treated with oral azithromycin, to inpatient departments to be treated with injectable antibiotics like carbapenems, thereby further burdening already struggling health systems in endemic regions. Moreover, with the dearth of novel antimicrobials in the horizon, we risk losing our primary defense against widespread mortality from typhoid. In addition to rolling out the WHO prequalified typhoid conjugate vaccine in endemic areas to decrease the risk of pan-resistant *Salmonella* Typhi strains, it is also imperative to implement antimicrobial stewardship and water sanitation and hygiene intervention to decrease the overall burden of enteric fever.

## Author summary

With the outbreak of extensively resistant (XDR) typhoid fever in Pakistan, azithromycin has become the last oral drug to treat typhoid. Although no azithromycin resistant XDR isolate has been reported to date, the increasing use of azithromycin and the clear historical record of widespread dissemination of resistance to all previously widely used antimicrobials by typhoidal *Salmonella*, suggest we will soon face strains resistant to all oral antibiotics. This makes it imperative to elucidate the mechanism of azithromycin resistance in typhoidal *Salmonella*. We tested 1,082 typhoidal *Salmonella* isolates from the two largest pediatric hospitals of Bangladesh and identified 13 azithromycin-resistant isolates. Using comparative genomics, we identified a mutation in a specific protein called AcrB that makes these isolates resistant to azithromycin. All azithromycin-resistant strains were susceptible to cephalosporin but resistant to all other oral antibiotics. The Pakistan outbreak strain is resistant to all common oral antibiotics and only susceptible to azithromycin. Acquisition of the plasmid that confers cephalosporin resistance in XDR strains by the Bangladeshi azithromycin-resistant strains or rise of the AcrB mutation in the XDR strains could be the end of oral treatment for typhoid. This poses serious threats to the health system of LMICs where typhoid is endemic. Currently, the majority of typhoid patients are prescribed oral treatment in the outpatient department, but an azithromycin-resistant XDR strain would shift enteric fever treatment from outpatient departments to inpatient departments to be treated with injectable antibiotics like carbapenems, further burdening already struggling health systems in endemic regions. Moreover, with the dearth of novel antimicrobials in the horizon, we risk losing our primary defense against widespread mortality from enteric fever.

## Introduction

Typhoid and paratyphoid, collectively known as enteric fever, are among the most common bacterial causes of morbidity worldwide, with the greatest burden in low- and middle-income countries [1]. *Salmonella enterica* subspecies *enterica* serovars Typhi (*Salmonella* Typhi) and Paratyphi (A, B and C), etiologies of enteric fever, cause an estimated 14 million illnesses and 136,000 deaths annually.

In the pre-antibiotic era, enteric fever mortality rates exceeded 30% in many areas, but ampicillin, chloramphenicol and co-trimoxazole were instrumental in reducing the rates to <1%. Resistance to all three antibiotics (referred to as multidrug resistance, MDR) emerged in late 1980’s [2], predominantly due to the rise and subsequent continental migration of H58 haplotype (now referred to as 4.3.1), which contained the resistance genes either on IncH1 plasmids or integrated within the chromosome [3–6]. Fluoroquinolones soon became the most-commonly prescribed antibiotic [7], but since the 2000’s there have been increasing reports of decreased fluoroquinolone susceptibility due to the acquisition of chromosomal mutations in the DNA gyrase and topoisomerase IV genes [8–13]. In Bangladesh, >99% of all Typhi and Paratyphi A strains exhibit decreased susceptibility to ciprofloxacin [14]. Based on the rising trends of fluroquinolone resistance, in 2011, WHO recommended ceftriaxone or azithromycin for treating *Salmonella* Typhi non-susceptible to fluoroquinolones [15].

The first report of ceftriaxone resistance emerged from Bangladesh in 2001, and since then there have been only sporadic reports of ceftriaxone-resistant *Salmonella* Typhi strains [16,17]. However, in 2016, an outbreak of extensively drug-resistant (XDR) *Salmonella* Typhi, resistant to chloramphenicol, ampicillin, cotrimoxazole, streptomycin, fluoroquinolones and third-generation cephalosporins was recognized in Pakistan and to date over 5000 cases have been confirmed [18,19]. Cephalosporin resistance of the XDR strains was caused by the acquisition of a broad-spectrum beta-lactamase resistance gene (bla-CTX-M-15) on an IncY plasmid found in *E*. *coli* isolates. Typhoid patients in Pakistan are primarily being treated in the out-patient department with the last available oral option, the macrolide azithromycin, resistance to which is uncommon [20]. This increasing use of azithromycin places selective pressure for the emergence and spread of azithromycin-resistant isolates, raising concerns of untreatable infections and increased mortality rates. Little is known about azithromycin resistance in typhoidal *Salmonella*; while there are some sporadic reports on azithromycin treatment failures [4,21–23], there are no data on the molecular mechanism of resistance.

In Bangladesh, *Salmonella* Typhi and Paratyphi A are the most common causes of bloodstream infections in children over 2 months of age and comprise of two-third of blood-culture positive isolates in microbiology laboratories [24]. Leveraging our surveillance system in place for enteric fever [25], here we describe the presence of azithromycin resistance among typhoidal *Salmonella* in Bangladesh and identify the molecular basis behind this resistance.

## Methods

### Study site and population

In this study, we report data from enteric fever surveillance conducted in the inpatient departments of the two largest pediatric hospitals of Bangladesh, Dhaka Shishu (Children) Hospital, DSH, and Shishu Shasthya (Child Health) Foundation Hospital, SSFH. These are sentinel sites of the World Health Organization supported Invasive Bacterial Vaccine Preventable Diseases surveillance platform in Bangladesh.

### Patient enrollment, etiology detection and antibiogram

Blood culture was performed at the discretion of the treating physicians. We enrolled patients with positive blood cultures for *Salmonella* Typhi or Paratyphi A. Blood cultures were performed using standard methods [24]. We aseptically obtained 2–3 milliliters of blood, which was inoculated into trypticase soy broth supplemented with sodium polyanethole sulphonate (0.25%) and isovitalex (1%). Incubated blood culture bottles were sub-cultured on the second, third, and fifth days of incubation. Identification of *Salmonella* Typhi/Paratyphi A isolates was confirmed using standard biochemical tests and agglutination with *Salmonella* species and serovar-specific antisera (Ramel, Thermo Fisher Scientific). Laboratory methods for blood culture and organism identification were consistent over the reporting period.

We used disc diffusion methods for determining antibiotic susceptibility patterns for azithromycin, ampicillin, co-trimoxazole, chloramphenicol, ciprofloxacin, levofloxacin, ceftriaxone, cefepime, cefixime and ceftazidime (Oxoid, Thermo Scientific, MA, USA). Azithromycin e-strips (bioMérieux, France) were used to determine the minimum inhibitory concentration (MIC) and confirm azithromycin resistance for strains that exhibited zone of clearance ≤12 mm in the presence of azithromycin discs. All results were interpreted according to the latest Clinical and Laboratory Standards Institute guidelines 2018 [26].

### DNA extraction and whole genome sequencing

We conducted whole genome sequencing on all identified azithromycin-resistant strains (12 *Salmonella* Typhi and 1 Paratyphi A). Isolates were grown on MacConkey agar (Oxoid, UK) overnight and DNA was extracted from a suspension of the overnight culture using the QIAamp DNA minikit (Qiagen, Hilden, Germany). Whole genome sequencing was performed on the Illumina HiSeq 4000 platform to generate 150 bp paired-end reads (Novogene Co. Ltd., Beijing, China). We used SPAdes 3.11.1 [27] to assemble the short paired-end reads into contigs for downstream analyses. All the sequences have been submitted to EnteroBase and NCBI (BioProject ID: PRJNA528114).

### Bioinformatics analysis

**Fig 1:**
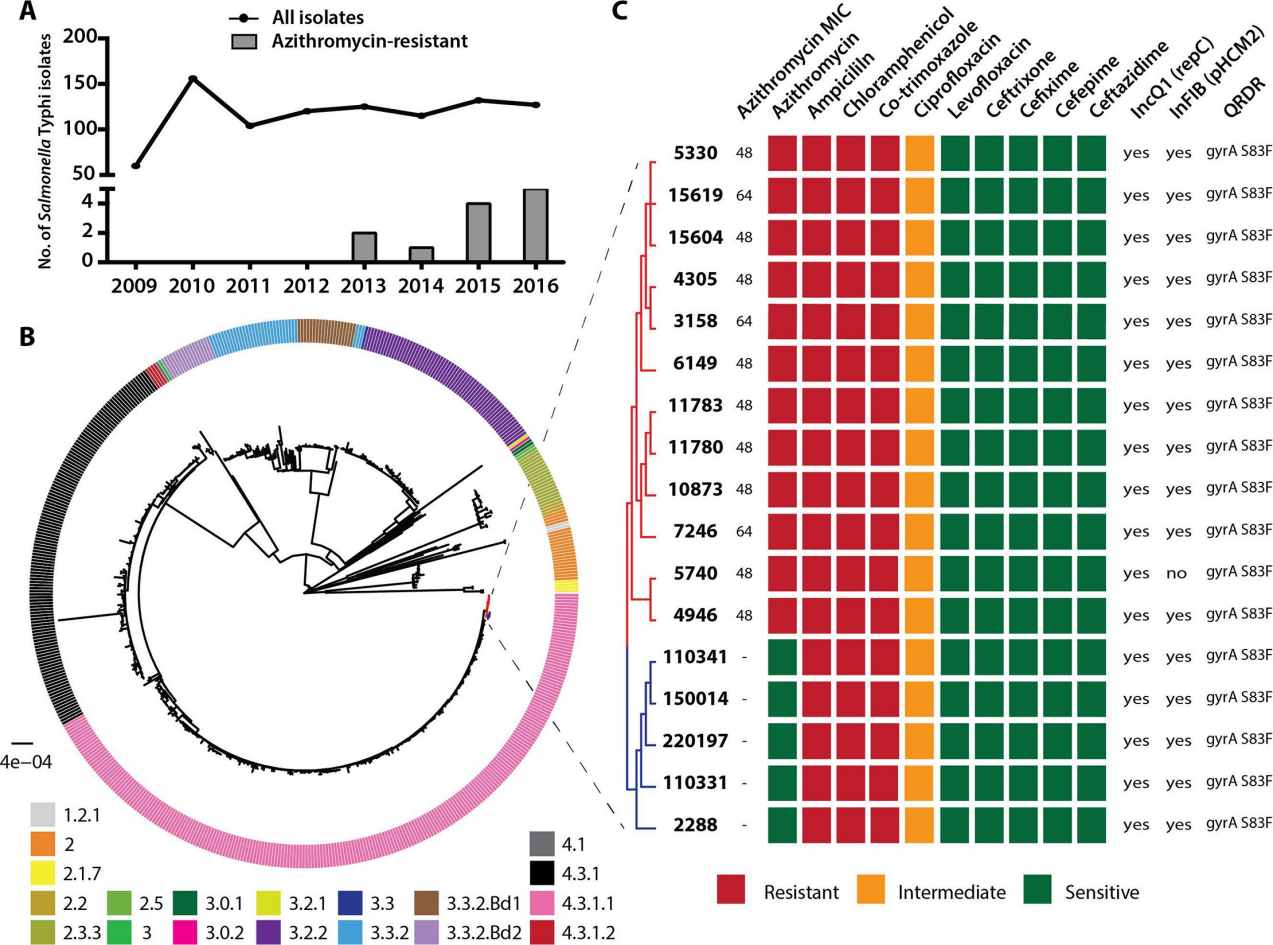
Emergence of azithromycin-resistant strains of *Salmonella* Typhi in Bangladesh and their genomic analysis. **(A)** Temporal distribution of 939 *Salmonella* Typhi isolates included in the study. The number of isolates is shown as the line plot from 2009–2016. The numbers of azithromycin-resistant strains isolated each year is shown in the bar plot. Azithromycin-resistant strains were first isolated in 2013. **(B)** Whole-genome SNP tree of 548 *Salmonella* Typhi strains isolated in Bangladesh including 12 strains from the present study and 536 strains from a previous study [28]. The tree highlights the different genotypes that are found in Bangladesh including the most prevalent genotype 4.3.1.1 (H58 lineage 1). The 12 azithromycin-resistant strains (colored in red) clustered together within the genotype 4.3.1.1. *Salmonella* Typhimurium strain LT2 was used as an outgroup, while *Salmonella* Typhi strain CT18 was used as the reference strain. **(C)** Predicted and experimentally determined antimicrobial susceptibility pattern of azithromycin-resistant *Salmonella* Typhi strains and the most-closely related five azithromycin-sensitive strains. The antimicrobial susceptibility was experimentally determined through disc diffusion assay against a panel of 10 antibiotics. The predicted transmissible elements and antimicrobial resistance markers are also shown.

For comparative genomic analysis, we compared the 12 azithromycin-resistant *Salmonella* Typhi strains with 536 strains that were previously isolated and genetically characterized by our group in Bangladesh [28]. Using the ParSNP tool [29], we constructed whole-genome SNP tree for 12 azithromycin resistant *Salmonella* Typhi strains with 536 strains from Tanmoy *et al*. [28]. *Salmonella* Typhi CT18 was used as the reference strain and *Salmonella* Typhimurium strain LT2 was used as the outgroup. SNPs present in genomic regions undergoing recombination were removed using the phipack package within the ParSNP tool. The final phylogenetic tree was obtained through RAxML (RAxML-NG v0.9.0) [30] with generalized time-reversible model and a Gamma distribution to model site-specific rate variation (GTR+ Γ substitution model) with 100 bootstraps to assess branch support. The genotypes were obtained using the Genotyphi script [6]. ggtree was used for tree visualization and overlaying the genotype data [31]. SRST2 0.2.0 [32], ResFinder [33] and CARD [34] were used to predict antimicrobial resistance markers, and PlasmidFinder [35] to identify the putative plasmids present in these strains. Finally, we compared the resistant strains to all sensitive *Salmonella* Typhi strains manually to find SNPs exclusive to the resistant strains (comparison to the most closely related 5 genomes are shown in Fig 1C) using the Gingr tool from the Harvest suite v1.1.2 [29]. To predict the function of the SNPs on protein function, we examined the protein structure (PDB ID: 3AOC) on PyMOL [36].

To determine the lineage of the azithromycin resistant *Salmonella* Paratyphi A strain 3144, we used 66 strains from Britto *et al*. [6] and 73 strains from Kuijpers *et al*. [37] that were found on Enterobase [38]. In this case, *Salmonella* Paratyphi A strain AKU_12601 was used as the reference strain and *Salmonella* Typhi strain CT18 was used as the outgroup. SNPs present in genomic regions undergoing recombination was removed using the phipack package within ParSNP tool. The final phylogenetic tree was obtained through RAxML (RAxML-NG v0.9.0) [30] with generalized time-reversible model and a Gamma distribution to model site-specific rate variation (GTR+ Γ substitution model) with 100 bootstraps to assess branch support. The lineage information for the 140 strains were obtained from Britto *et al*. [6] and Kuijpers *et al*. [37], and ggtree was used to visualize the phylogenetic tree and overlay the lineage information.

### Macrolide susceptibility test in *E*. *coli* and *Salmonella* Typhi

We amplified the *acrB* genes from azithromycin-resistant *Salmonella* Typhi strain 5003 (SAMN11174925) and Paratyphi A strain 3144 (SAMN11174919) and azithromycin-sensitive Typhi strain 4119 and Paratyphi A strain 4071 using PCR with forward and reverse primers with overhangs complementary to pHERD30T (TAAAACGACGGCCAGTGCCAAGCTTTCAGCGATGTTCTGTCGAATGAC and TAAAACGACGGCCAGTGCCAAGCTTTCAATGATGATCGACAGTATGGCTG respectively). The genes were inserted into the multiple cloning site of pHERD30T using Gibson assembly [39]. We verified the sequences of all inserted genes through Sanger sequencing. The plasmids were chemically transformed into CaCl_2_-competent *E*. *coli* BW25112 Δ*acrB* strains obtained from the Keio collection [40], and electroporated into Typhi strain 4119 and Paratyphi A strain 4071. *E*. *coli* strains with plasmids (with or without an insert) were tested for susceptibility patterns for erythromycin, azithromycin, ampicillin, co-trimoxazole, chloramphenicol, ciprofloxacin, levofloxacin, ceftriaxone, cefepime, cefixime and ceftazidime using the disc diffusion method, and MIC was determined using azithromycin E-strips. Typhi and Paratyphi A strains with plasmids (with or without an insert) were tested for susceptibility patterns for azithromycin using E-strips.

### Ethical clearance

The protocols were approved by the ethics review committees of the Bangladesh Institute of Child Health, DSH. Blood samples were collected and received at the laboratory as part of routine clinical care and informed written consent was obtained from parents or caregivers for other aspects of the study, including data collection and use of specimens for additional laboratory analysis.

## Results

### Emergence of azithromycin-resistant *Salmonella* Typhi and Paratyphi A

Between 2009 and 2016, through our enteric fever surveillance [24] in the inpatient departments of the two largest pediatric hospitals of Bangladesh, we isolated 939 *Salmonella* Typhi and 143 Paratyphi A strains. Twelve of the Typhi and one of the Paratyphi A strains were resistant to azithromycin, with disc diameters of ≤12 mm, and minimum inhibitory concentration (MIC) of ≥32 μg/ml [41]. All 12 azithromycin-resistant *Salmonella* Typhi strains were also MDR and were increasingly isolated since 2013 (Fig 1A), marking gradual emergence of azithromycin-resistant *Salmonella* Typhi in Bangladesh. All patients with azithromycin-resistance typhoid or paratyphoid lived in Dhaka city, known to be endemic for enteric fever [14,24,25,42] (S1 Fig).

### Azithromycin resistant *Salmonella* Typhi harbors a mutation in the AcrB efflux pump

We sequenced the 12 azithromycin-resistant Typhi strains and found that all azithromycin-resistant strains belonged to genotype 4.3.1.1 (H58 lineage 1), the most common genotype found in Bangladesh [4,28]. In a whole-genome single nucleotide polymorphism (SNP) tree, the 12 strains clustered together indicating that they are genetically similar to one another and potentially arose for a single common ancestral strain (Fig 1B, S1 Table). To identify the genetic basis of azithromycin resistance, we used three bioinformatic tools: SRST2 [32], Resfinder [33] and CARD [34] and to evaluate the results obtained from these tools, we tested antimicrobial susceptibility against a panel of nine other antibiotics (Fig 1C, S1 Table). While the tools successfully predicted the observed susceptibility patterns for the nine antibiotics, no known azithromycin resistance mechanism was identified (Fig 1C). Using PlasmidFinder [35] we identified two mobile genetic elements in these *Salmonella* Typhi strains: (i) repC (12/12 strains), a ~24kbp Tn2670-like or SGI11-like complex transposable element carrying AMR genes which is found commonly in isolates and is integrated into the chromosome [4], and (ii) IncFIB (11/12 strains) on plasmid pHCM2 commonly found in *Salmonella* Typhi strains but not known to harbor AMR genes [43,44]. Both these mobile elements were also present in closely related azithromycin-sensitive strains. The lack of known azithromycin-resistance genes indicated a novel mechanism of azithromycin resistance in these strains.

**Fig 2:**
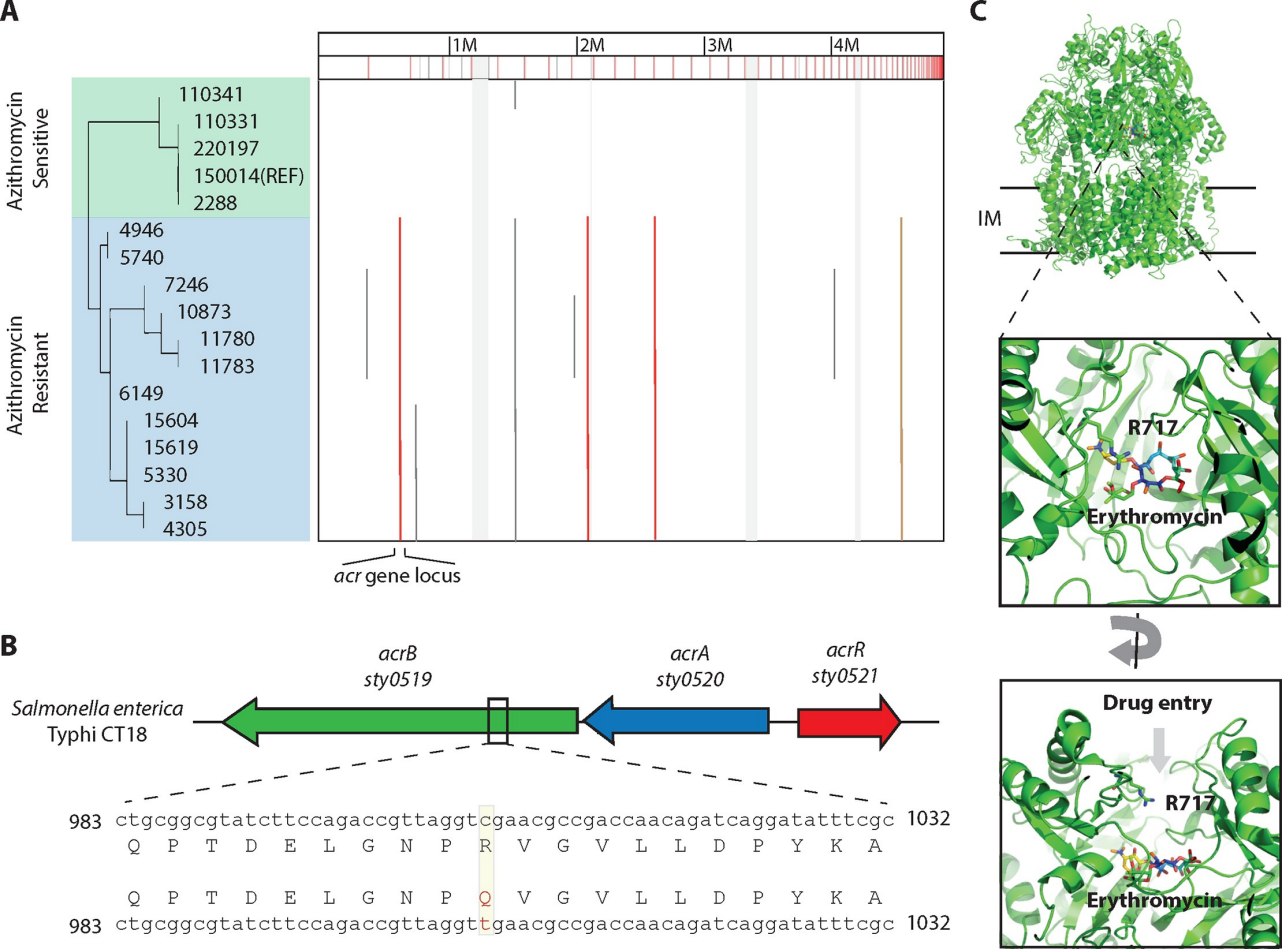
Identification of R717Q mutation on AcrB efflux pump as a cause of azithromycin resistance in *Salmonella* Typhi. **(A)** Whole genome sequence alignment of 12 azithromycin-resistant and 5 most genetically similar azithromycin-sensitive *Salmonella* Typhi strains (identified in the phylogenetic tree in Fig 1B). Whole genome SNP detection and alignment was done using ParSNP and results were visualized in Gingr [29]. The single nucleotide polymorphisms (SNPs) unique to the resistant strains are highlighted with vertical lines. Four SNPs were identified: 3 non-synonymous (shown as a red line) and 1 synonymous SNPs (shown as a green line) that are exclusive to the azithromycin resistant strains. **(B)** The *acr* gene cluster in *Salmonella* Typhi reference strain CT18. One of the SNPs found exclusively in azithromycin-resistant strains was mapped to the gene cluster composed of: *acrA* (STY0520) and *acrB* (STY0519), that encodes a periplasmic and inner membrane protein component of the RND-efflux pumps respectively, and *acrR* (STY0521), a transcriptional regulator of AcrA/B protein synthesis. The SNP was present on the *acrB* gene and resulted in the change of an arginine (R) at position 717 to a glutamine (Q) residue on the encoded AcrB protein (highlighted in yellow). **(C)** R717Q mutation is present at the periplasmic cleft of the proximal binding pocket on AcrB. Structure of *E*. *coli* AcrB (PDB ID: 3AOC) is shown in green with the macrolide erythromycin bound in the proximal drug binding pocket. AcrB is present in the inner membrane (IM) of the bacterial cells and drug molecules, including macrolides, enter the AcrB pump through a periplasmic opening that leads to a proximal binding pocket. The drug molecules are shuttled outside the cells through the proximal binding pocket with the help of the proton motive force. R717 lines the entry of the periplasmic opening.

We compared the sequences of these 12 azithromycin-resistant strains to that of 536 Typhi strains that we had previously sequenced and genetically characterized [28]. In the WGS SNP tree, we identified four unique SNPs, present only in the 12 azithromycin-resistant strains, three of which were non-synonymous: STY2741 (codes for PurN, a glycinamidine ribonucleotide transformyltransferase), STY1399 (codes for a hypothetical protein) and STY0519 (codes for AcrB, an inner membrane permease) (Fig 2A, S2 Fig). For the first two candidates, there is no evidence of their involvement in mediating antimicrobial resistance in the literature. However, the third gene, *acrB* is part of a trans-envelope resistance-nodulation-division (RND) efflux pump that has been previously reported to transport macrolides including azithromycin across the bacterial cell envelope, making it the most promising candidate [45]. Mutations affecting expression of AcrB have been implicated in macrolide resistance in *Neisseria gonorrhoeae* [46]. Furthermore, laboratory mutagenesis studies in *Escherichia coli* have shown that mutations in *acrB* can lead to higher macrolide efflux thereby contributing to resistance [47]. The SNP observed in the 12 azithromycin-resistant *Salmonella* Typhi strains changed the arginine residue (R) at position 717 to a glutamine (Q) (Fig 2B). R717 is a conserved residue on the periplasmic cleft that acts as the entry portal for most drugs in AcrB (Fig 2C).

### R717 mutations in AcrB confer azithromycin resistance

**Fig 3:**
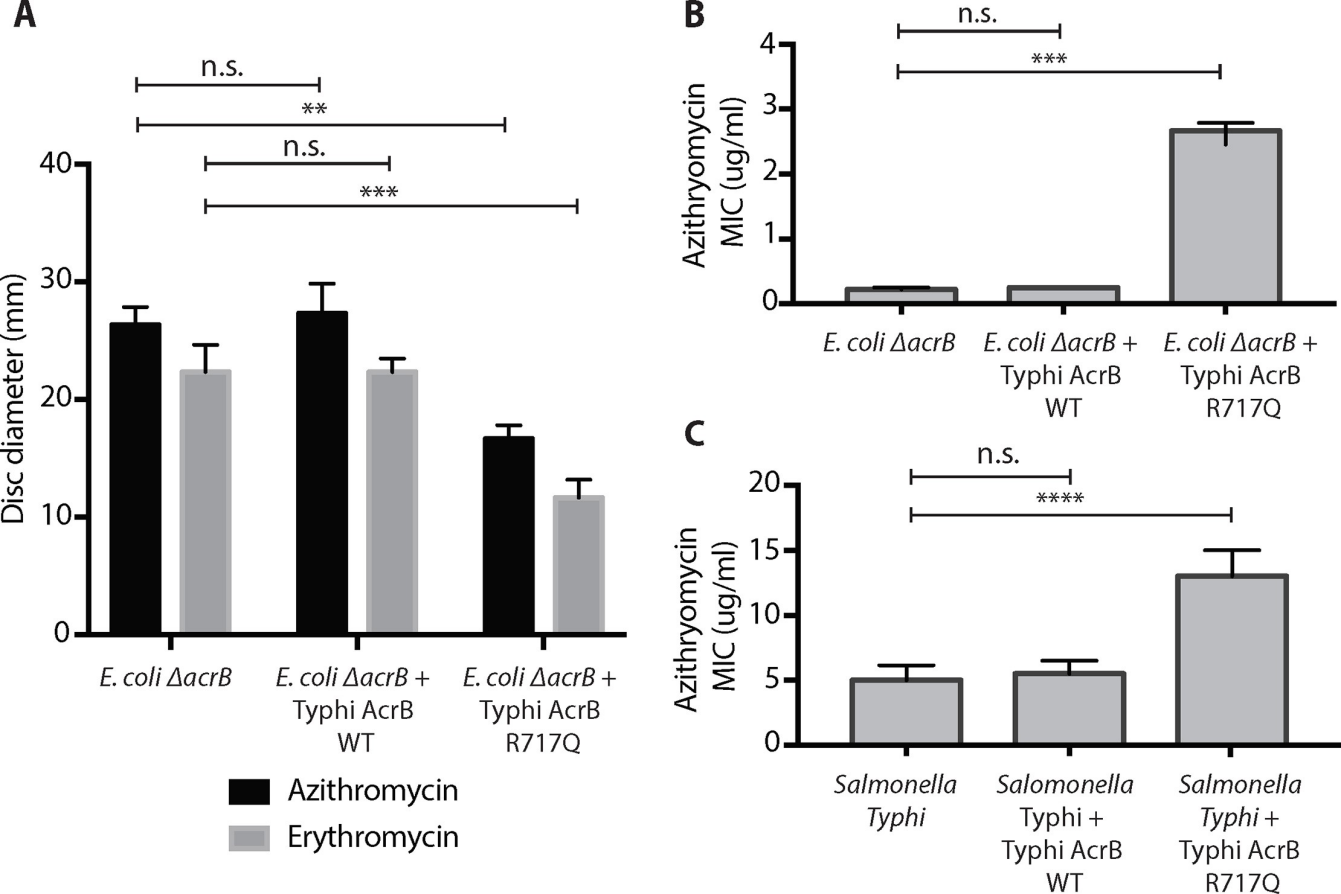
AcrB R717Q increases efflux of macrolides in *E*. *coli* and *Salmonella* Typhi strains. Quantification of results obtained from three biological replicates for **(A)** disc diffusion and **(B)** E-strip assays are shown for *E*. *coli* BW25113 *ΔacrB* transformed with different plasmids. **(C)** Quantification of results obtained from at least three biological replicates from azithromycin E-strip assay in Typhi strain 4119. One way-ANOVA with multiple comparisons was used to test statistical significance. ns: p>0.05; **: p ≤ 0.01; ***: p ≤ 0.001, ****: p ≤ 0.0001.

We cloned *acrB* from azithromycin susceptible and resistant *Salmonella* Typhi strains into an *E*. *coli* plasmid and introduced them into an *E*. *coli* strain that lacks the endogenous *acrB* (*E*. *coli ΔacrB*). Compared to *E*. *coli* strains containing empty plasmid or wild type *acrB*, the strain expressing AcrB-R717Q showed a smaller zone of disc clearance for both azithromycin (26.3 mm vs 16.7 mm, *p* = 0.0013) and erythromycin discs (22.3 mm vs 11.7 mm, *p value* = 0.0009) and exhibited a 11-fold increase in azithromycin MIC (0.22 μg/ml vs 2.7 μg/ml, *p* = 0.0002) (Fig 3A and 3B). Certain AcrB mutations have previously been shown to effect transport of other antibiotics such as ciprofloxacin [48], but the R717Q mutation did not change the susceptibility patterns for any other nine antibiotics we tested, indicating the specificity of this mutation towards azithromycin resistance (S2 Table). For further confirmation of the effects of this mutation in *Salmonella* Typhi, we introduced the plasmids in an azithromycin-sensitive Typhi strain and observed a 3-fold increase in MIC (5 μg/ml vs 13 μg/ml, *p* < 0.0001) in the presence of AcrB-R717Q (Fig 3C). The difference here is lower compared to that seen in *E*. *coli ΔacrB* plausibly because the Typhi strain contains endogenous wild-type AcrB competing against the exogenous AcrB-R717Q that we artificially introduced. Taken together, these results confirm that AcrB-R717Q specifically leads to increased macrolide resistance.

**Fig 4:**
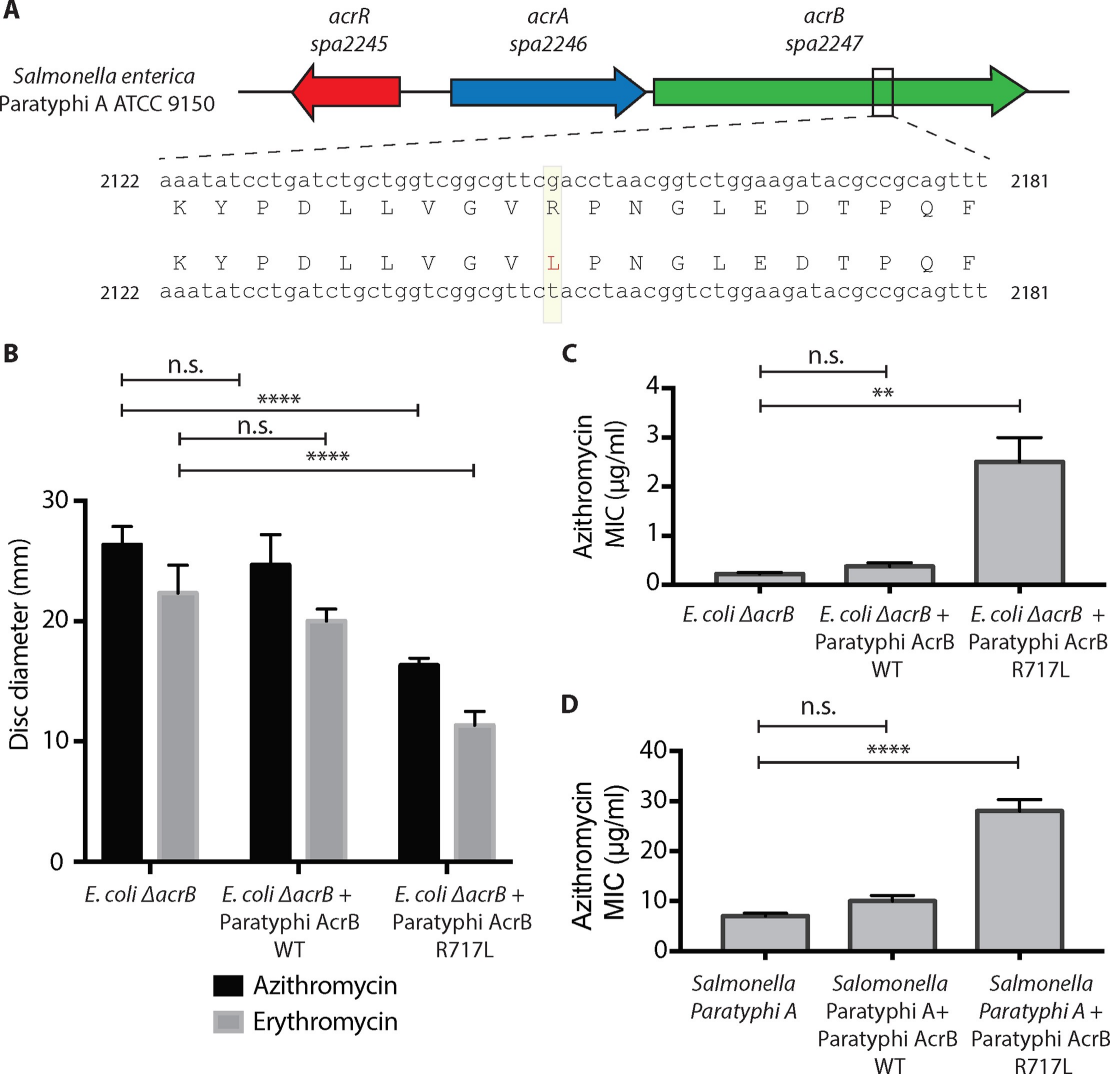
Identification of R717L mutation in AcrB protein in *Salmonella* Paratyphi A strains. **(A)** Sequence alignment of *acrB* gene from the azithromycin-resistant *Salmonella* Paratyphi A strain to the *acrB* gene (*spa2247*) in the reference strain ATCC 9150. A SNP was identified that changed the R717 to a leucine (L) residue (highlighted in yellow). Quantification of results obtained from three biological replicates for **(B)** disc diffusion and **(C)** E-strip assays in *E*. *coli* are shown. **(D)** Quantification of results obtained from at least three biological replicates from azithromycin E-strip assay in Paratyphi A strain 4071 is shown. One way-ANOVA with multiple comparisons was used to test statistical significance. ns: p>0.05; **: p ≤ 0.01; ****: p ≤ 0.0001.

We conducted an extensive BLAST search to identify other typhoidal *Salmonella* strains with mutations in AcrB the NCBI database and found only one *Salmonella* Typhi strain isolated in Oceania in 2008 (Typhi_10349_1#30, genotype 3.5.4, a non-MDR strain that contains *gyrA* D87N) that contained the same R717Q mutation, however no AMR data were available for this strain. Interestingly, whole genome sequencing of the one azithromycin-resistant *Salmonella* Paratyphi A strain (lineage C4, S3 Fig) identified during our surveillance showed that this strain also contained a mutation in *acrB* which changed R717 to a leucine (L) (Fig 4A). This mutation was absent in the genomes of the Paratyphi A strains in the NCBI database. To determine the effect of R717L mutation, we expressed Paratyphi A wild-type AcrB and AcrB-R717L in *E*. *coli ΔacrB* (Fig 4B and 4C). As seen for Typhi AcrB R717Q, Paratyphi A AcrB-R717L leads to a smaller disc clearance for azithromycin (26.3 mm vs 16.3 mm, *p* = 0.0001) and erythromycin (22.4 mm vs 11.4 mm, *p* = 0.0001) and 10-fold higher azithromycin MIC (0.22 μg/ml vs 2.5 μg/ml, *p* = 0.003). When these plasmids were introduced in a sensitive Paratyphi A strain, we observed 4-fold change in MIC (7 μg/ml vs 28 μg/ml, *p* = 0.0001) in the presence of the R717L mutation, confirming that mutations in R717 lead to macrolide resistance.

## Discussion

Rising antimicrobial resistance threatens the progress made so far in management of enteric fever. In this study, we report the gradual rise of resistance amongst typhoidal *Salmonella* in Bangladesh to azithromycin, the last available oral antibiotic to treat enteric fever. We identified 12 azithromycin-resistant *Salmonella* Typhi strains and one Paratyphi A strain. The molecular basis of this resistance is one mutation in the RND efflux pump AcrB protein at position 717 and this is the first report demonstrating the impact of this mutation in conferring macrolide resistance in a clinical setting.

AcrB has not been previously implicated in macrolide resistance in *Salmonella*. However, previous work has shown that mutations of AcrB or changes in its expression can lead to resistance in *Neisseria gonorrhoeae* and *Escherichia coli*, but the specific mutation at position 717 is not predicted to confer antibiotic resistance in any available antibiotic resistant prediction tools [46,47]. The SNP we observed in the 12 azithromycin-resistant *Salmonella* Typhi strains changed the arginine residue (R) at position 717 to a glutamine (Q), while in the azithromycin-resistant *Salmonella* Paratyphi A strain the same arginine residue was changed to a leucine (L). The arginine at position 717 is a conserved residue on the periplasmic cleft that acts as the entry portal for most drugs in AcrB. Change of a large positively charged arginine to a smaller glutamine or leucine may affect the movement of macrolides into the drug pocket, which in turn may affect their subsequent efflux via AcrB. Interestingly, in a previous mutagenesis study, substitution of the arginine residue with an alanine (R717A) was found to partially increase efflux of the macrolide erythromycin in *E*. *coli* [49]. Furthermore, a recent study using molecular dynamics simulation showed that AcrB R717L mutation increases the uptake of macrolides into the drug pocket, increasing azithromycin resistance in *Salmonella* Typhimurium in vitro [50].

Azithromycin is widely used empirically in hospitals and sold over the counter in Bangladesh since 1995. Azithromycin and third generation cephalosporins like cefixime are currently the most common empirical drug used for treatment of febrile illnesses including enteric fever in the out-patient departments and community. Although the rate of azithromycin resistance of typhoidal *Salmonella* in Bangladesh is low and the genetic basis is a chromosomal SNP, in light of the outbreak of XDR typhoid in Pakistan, increased azithromycin use can place selective pressure on strains such as the ones with R717 mutations to spread. Although no azithromycin resistant XDR isolate has been reported to date, the increasing use of azithromycin and the clear historical record of widespread dissemination of resistance to all previously widely used antimicrobials by *Salmonella* Typhi and Paratyphi A [3,4,44] suggest we will soon face strains resistant to almost all oral antibiotics.

There have been two reports of ceftriaxone-resistant *Salmonella* Typhi from Bangladesh to date [16,17]. Acquisition of the plasmid that confers ceftriaxone resistance in XDR strains by the Bangladeshi azithromycin-resistant strains will bring us to brink of the end of oral treatment for typhoid. Similarly, the rise of a point mutation like R717Q in AcrB in the XDR Typhi outbreak strain of Pakistan will be catastrophic. In endemic countries like Bangladesh and Pakistan, typhoidal *Salmonella* is the primary etiology of bloodstream infections. Currently the majority of typhoid patients are prescribed oral treatment in the outpatient department and sent home, but an azithromycin-resistant XDR strain would shift enteric fever treatment from outpatient departments, to inpatient departments to be treated with injectable antibiotics like carbapenems, thereby further burdening an already struggling health systems in endemic regions [51,52]. For example, in Dhaka Shishu Hospital, each year 23,000 children are admitted, and about 6,000 children are refused admission because of lack of available beds, despite being the largest pediatric hospital [52]; hundreds of thousands of children are treated in the out-patient department and sent home. This situation will be much worsened if the option for out-patient based oral treatment of typhoid is not available.

The findings of this study should be considered within the context of a few limitations. The proportion of azithromycin resistance in typhoidal *Salmonella* in this study was 1.2% (13 of 1,082), and the strains used in this study were derived from a tertiary-level hospital in-patient setting. Thus, it is not possible to predict the proportion of azithromycin resistance cases in the community, where most typhoid cases are treated. Azithromycin resistance rates would also differ in other countries, or between rural vs urban sites within Bangladesh. Furthermore, even though we followed the latest CLSI guidelines [34], we are unable to assess the clinical implication of this mutation as comprehensive clinical and treatment history of the patients was not available. On the other hand, there have been reports of azithromycin treatment failure, but there is no genomic data to further investigate those cases.

With the dearth of novel antimicrobials on the horizon, we risk losing our primary defense against widespread mortality from enteric fever and falling back into the pre-antibiotic era. In 2018, the first typhoid conjugate vaccine was prequalified, and endemic countries are now facing important decisions regarding its introduction. Considering the high burden of typhoid, and the rising AMR crisis, introduction of the vaccine is speculated to decrease the burden, which in turn, is expected to reduce AMR infections and thus overall use of antibiotics as typhoid is the most common cause of bloodstream infection infections in endemic countries [51]. However, the vaccine does not protect against paratyphoid, for which AMR is an equally important issue to address. In addition to the roll-out of the vaccine in endemic areas, introduction of water, sanitation and hygiene interventions, antibiotic stewardship and continued AMR surveillance will also be very important to decrease the overall burden of enteric fever and tackle the arms race against rising resistance.

## Supporting information

S1 Fig Spatiotemporal distribution of azithromycin-resistant *Salmonella* Typhi and Paratyphi A strains. The 13 azithromycin-resistant typhoidal *Salmonella* strains were isolated from Dhaka Shishu Hospital (shown in red). All the patients lived within the Dhaka municipal area. The map was made using the R packages maptools and raster.
(TIFF)

S2 Fig Genetic and structural analysis of 2 other non-synonymous SNPs. **(A)** SNP on *sty2741* gene (also known as *purN*) that encodes a glycinamidine ribonucleotide transformyltransferase (GAR-Tfase) enzyme. The SNP leads to change in W195 to a stop codon, leading to premature termination of the protein sequence (highlighted in yellow). The structure of *E*. *coli* GAT-Tfase (green, PDB ID: 1C3E) in complex with an inhibitor (shown in red) highlighting the active site is shown. The W195 is present close to the C-terminus and premature termination at this position is predicted to not affect protein function **(B)** SNP on *sty1399* that encodes a hypothetical protein that is proposed to contain a B3/B4 tRNA-binding domain. The function of this protein is not known and the SNP results in conversion of an alanine residue at position 34 to a threonine residue (highlighted in yellow). None of these two genes have been previously implicated in macrolide resistance.
(TIFF)

S3 Fig *Salmonella* Paratyphi A strain 3144 belongs to lineage C4. To identify the lineage of azithromycin resistant strain 3144, it was compared to 139 Paratyphi A strains from different parts of the world. Whole genome SNP tree was made using RAxML and visualized in ggtree. The lineage information was obtained from Britto *et al*. [6] and Kuijpers *et al*. [37] and shown as the ring around the tree.
(TIFF)

S1 Table Characteristics of *Salmonella* Typhi and Paratyphi A isolates reported in the study. (XLSX)

S2 Table AcrB mutations do not affect efflux of other families of antibiotics. Susceptibility of *E*. *coli* strains with empty, *Salmonella* Typhi AcrB WT/R717Q and Paratyphi A AcrB WT/R717L were tested against a panel of 9 different antibiotic including 5 beta-lactams, 2 fluoroquinolones, 1 phenicol and 1 diaminopyrimidine /sulphonamide. The data are shown as mean and standard error from 3 different biological replicates.
(XLSX)
