# Supplementary figures and images for: Molecular mechanism of azithromycin resistance among typhoidal Salmonella strains in Bangladesh identified through passive pediatric surveillance
Source: PLoS Negl Trop Dis. 2019 Nov 15;13(11):e0007868. doi: 10.1371/journal.pntd.0007868 (PMC6881056; doi:10.1371/journal.pntd.0007868)

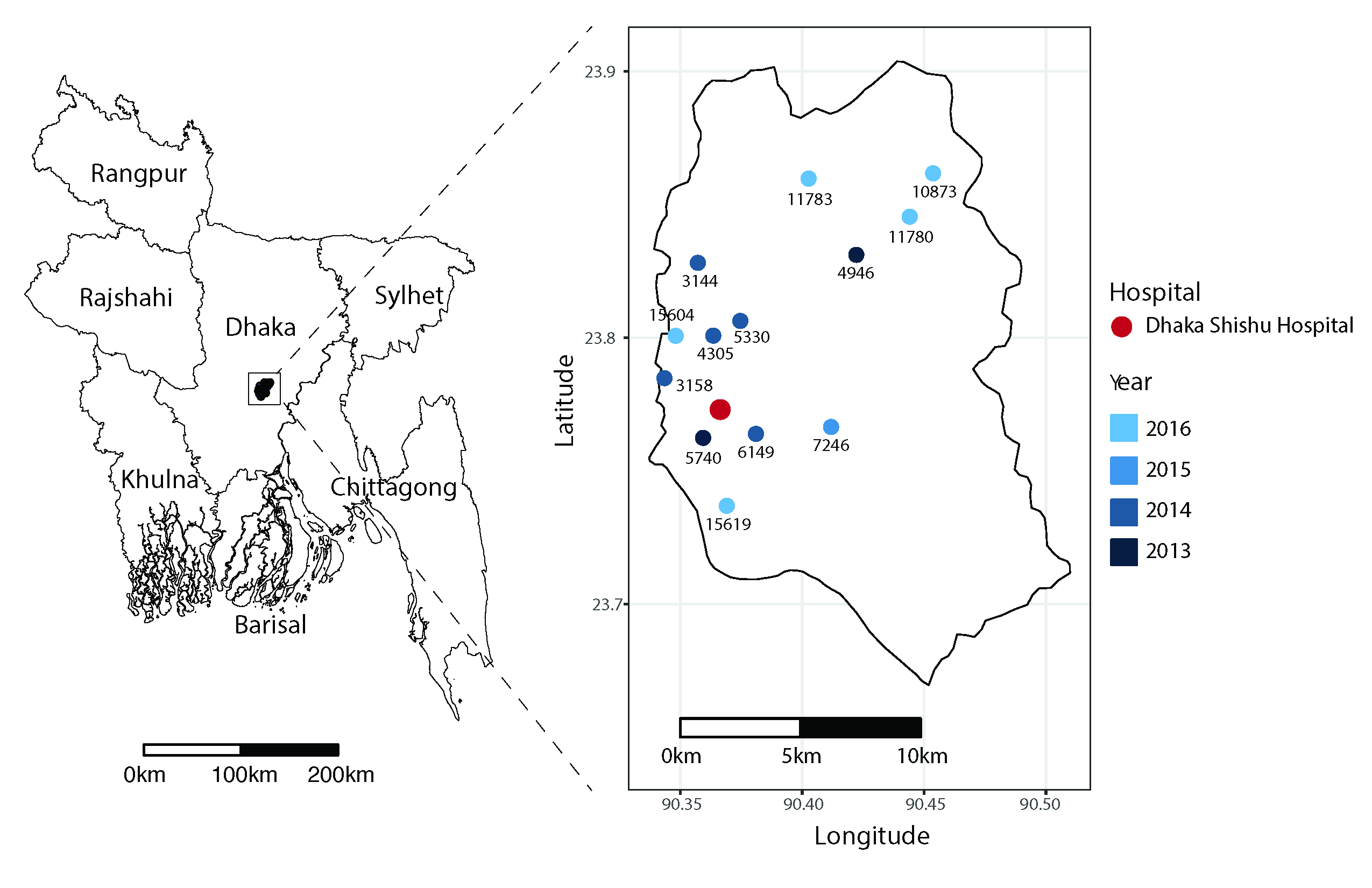

Supplement: S1 Fig — The 13 azithromycin-resistant typhoidal Salmonella strains were isolated from Dhaka Shishu Hospital (shown in red). All the patients lived within the Dhaka municipal area. The map was made using the R packages maptools and raster. (TIFF) [file pntd.0007868.s001.tiff]

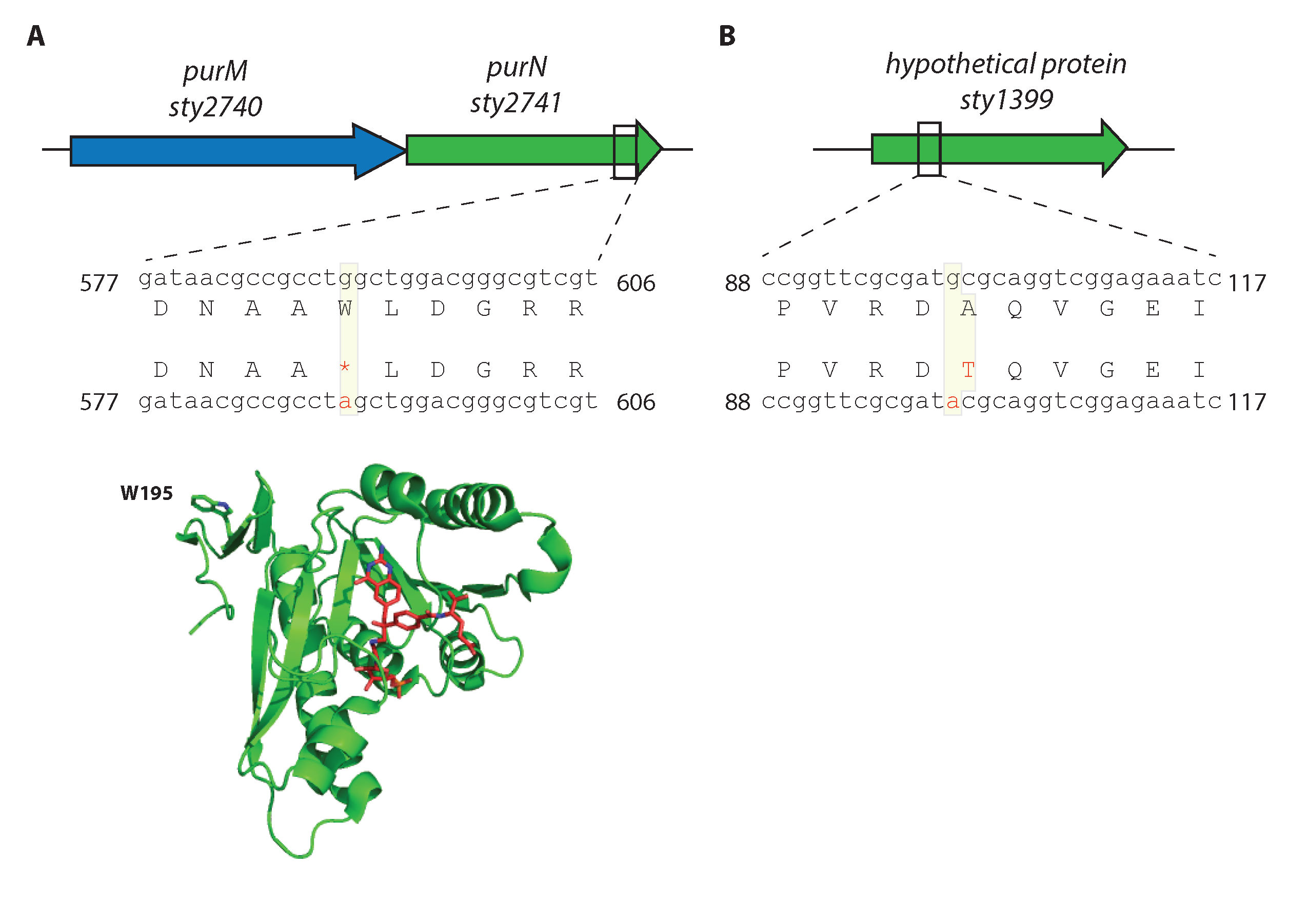

Supplement: S2 Fig — (A) SNP on sty2741 gene (also known as purN) that encodes a glycinamidine ribonucleotide transformyltransferase (GAR-Tfase) enzyme. The SNP leads to change in W195 to a stop codon, leading to premature termination of the protein sequence (highlighted in yellow). The structure of E. coli GAT-Tfase (green, PDB ID: 1C3E) in complex with an inhibitor (shown in red) highlighting the active site is shown. The W195 is present close to the C-terminus and premature termination at this position is predicted to not affect protein function (B) SNP on sty1399 that encodes a hypothetical protein that is proposed to contain a B3/B4 tRNA-binding domain. The function of this protein is not known and the SNP results in conversion of an alanine residue at position 34 to a threonine residue (highlighted in yellow). None of these two genes have been previously implicated in macrolide resistance. (TIFF) [file pntd.0007868.s002.tiff]

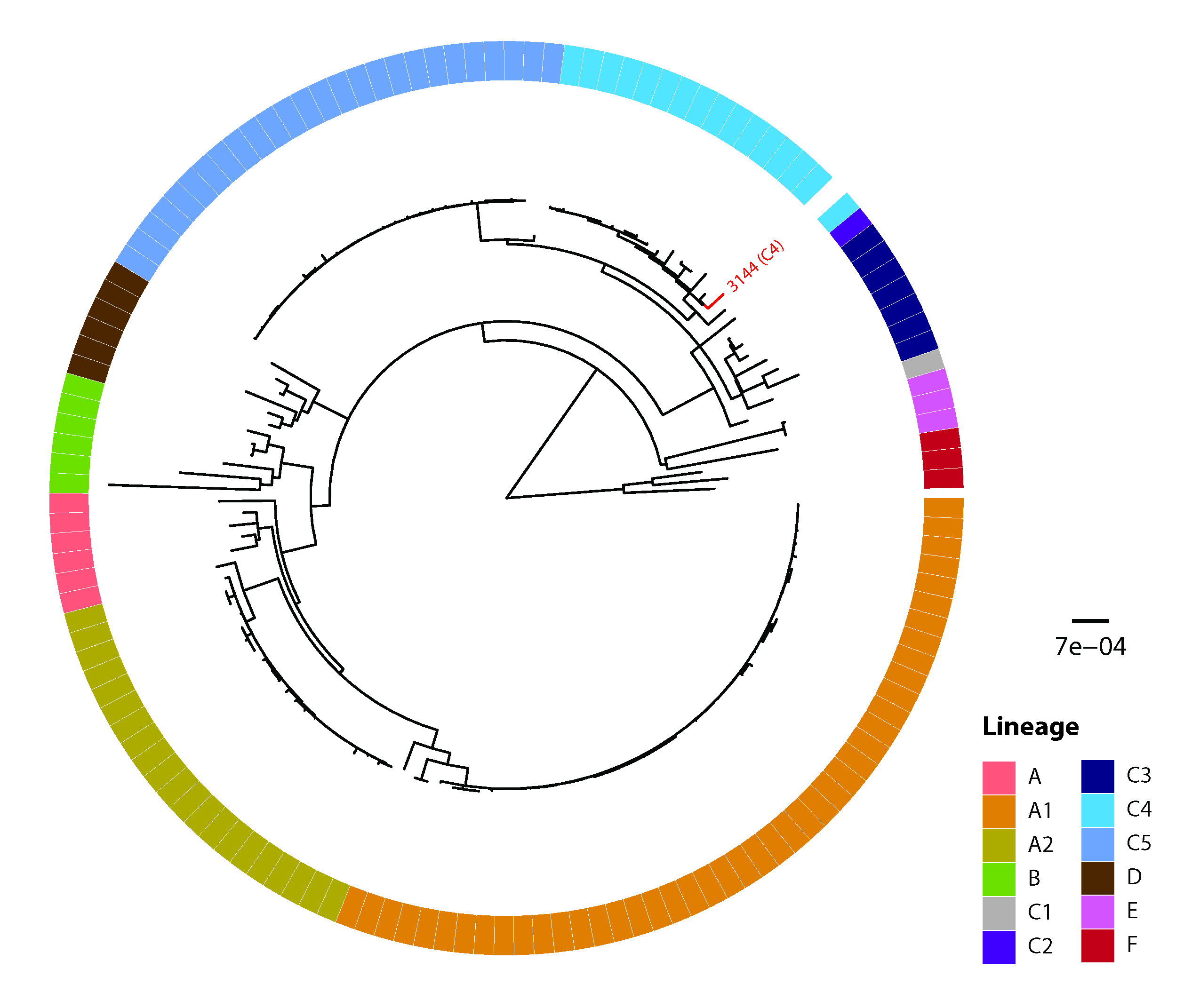

Supplement: S3 Fig — To identify the lineage of azithromycin resistant strain 3144, it was compared to 139 Paratyphi A strains from different parts of the world. Whole genome SNP tree was made using RAxML and visualized in ggtree. The lineage information was obtained from Britto et al. [6] and Kuijpers et al. [37] and shown as the ring around the tree. (TIFF) [file pntd.0007868.s003.tiff]
